# Cardiovascular function is not associated with creatine kinase activity in a black African population: The SABPA study

**DOI:** 10.1186/s12872-016-0315-2

**Published:** 2016-06-10

**Authors:** Catharina M. C. Mels, Caitlynd van Zyl, Hugo W. Huisman

**Affiliations:** Hypertension in Africa Research Team (HART), North-West University, Potchefstroom Campus, Private Bag X6001, Potchefstroom, 2520 South Africa; MRC Research Unit for Hypertension and Cardiovascular Disease, North-West University, Potchefstroom, South Africa

**Keywords:** Blood pressure, Creatine kinase activity, Ethnicity, Pulse pressure, Total peripheral resistance

## Abstract

**Background:**

Higher creatine kinase (CK) activity is associated with the development of cardiovascular disease in black African populations. We compared CK activity and investigated associations of blood pressure with CK activity in black and white men as well as black and white women.

**Methods:**

Ambulatory blood pressure, total peripheral resistance and pulse wave velocity of 197 black and 208 white participants were determined and serum CK activity was measured.

**Results:**

Blood pressure and pulse wave velocity were higher in black men and women (all *p* < 0.001) when compared to their white counterparts. CK activity only varied between black and white women (75.9 U/l vs 62.8 U/l, *p* = 0.009), even after adjusting for age, body mass index and physical activity. Despite the worse cardiovascular profile of black men and women, and the higher CK activity in the black women, we were unable to link blood pressure, pulse wave velocity or total peripheral resistance with CK activity, in the black African population. In white men, total peripheral resistance was associated with CK activity (*R*^2^ = 0.32; β = 0.25; *p* = 0.009), whereas systolic blood pressure (*R*^2^ = 0.46; β = 0.17; *p* = 0.03) and pulse pressure (*R*^2^ = 0.31; β = 0.21; *p* = 0.01) were associated with CK activity in white women.

**Conclusions:**

The lack of associations in the black African population suggests that the link between a worse cardiovascular profile and CK activity may be overshadowed by other contributing factors. Whereas, the established link between cardiovascular function and CK activity in the white groups may be the result of enhanced smooth muscle cell contractility and/or attenuated nitric oxide synthesis capacity.

**Electronic supplementary material:**

The online version of this article (doi:10.1186/s12872-016-0315-2) contains supplementary material, which is available to authorized users.

## Background

While the prevalence of cardiovascular disease is much higher in black African populations than in white populations, it is the topic of many research questions [1–3]. Creatine kinase (CK), an enzyme responsible for the rapid release of adenosine triphosphate, has higher activity in all tissues with high and/or changing energy demands (brain, heart, renal artery and skeletal muscle) in black participants compared to white participants of sub-Sahara African descent [4]. As a result of the observed higher CK activity in black populations, it has been suggested that this may be the missing link to explain the higher prevalence of cardiovascular disease in black populations [5].

In another study the association of increasing blood pressure with higher CK activity was not limited to a certain ethnic group, since the study population included descendants of Africa, South Asia and white Europeans [6]. The same relationship was also established in in a normal Norwegian white population [7]. Furthermore even slight elevations in serum CK levels are associated with increased risk for myocardial infarction especially in individuals with dyslipidemia [8].

Additional to the higher CK activity in black populations, CK activity also differs between gender groups. Substantial heterogeneity in CK activity were observed in a black and white study population which included both gender groups, resulting in the establishment of different reference ranges for black men (52–520 U/l), white men and black women (35–345 U/l) and white women (25–145 U/l) [9].

Recent South African based evidence indicated that behavioural risk markers are the main contributing factors to the 24 % increase in hypertension incidence, in participants followed for 5 years [2]. These factors included abdominal obesity and elevated γ-glutamyl transferase (γ-GT) (as marker of alcohol abuse), which plays an important role in the metabolism of glutathione. Furthermore, elevated γ-GT levels also predicted cardiovascular and all-cause mortality [10] while psychological distress predicted the development of hypertension over 5 years, even after adjusting of γ-GT levels [11]. Taken together, these results suggests that behavioural risk factors and psychological distress are to blame for the worse cardiovascular function observed in the black population of sub-Sahara Africa, whereas other studies suggest higher CK activity to be the culprit.

The aim of our study was therefore to compare CK activity between black and white men and women. We further aimed to investigate if ambulatory systolic blood pressure (SBP) and diastolic blood pressure (DBP) are associated with CK activity in black and white men as well as black and white women.

## Methods

### Study population and protocol

This study is embedded in the Sympathetic activity and Ambulatory Blood Pressure in Africans (SABPA) study, a cross-sectional target population study aimed to investigate neural mechanistic pathways involved in emotional distress and vascular remodelling. The study population and protocol has been described elsewhere [12]. Briefly, 101 black and 101 white male and 99 black and 108 white female teachers from the Dr. Kenneth Kaunda Education District in the North West Province of South Africa between the ages of 20 and 65 years were included in the study. Exclusion criteria included pregnancy, lactation, elevated ear temperature (>37 °C), and blood donation or vaccination 3 months prior to the commencement of the study.

For this sub-study we also excluded participants with missing CK activity values, leaving a total of 197 black and 208 white participants. Participants were fully informed about the objectives and procedures of the study prior to their recruitment and informed consent was obtained from the selected participants prior to commencement of the study. The study was conducted in line with the ethical principles of the Declaration of Helsinki 1975 (last updated in 2013) and was approved by the Ethics Review Board of the North-West University (Potchefstroom Campus) (NWU-00036-07-S6).

On the first day at 07:00 the ambulatory blood pressure monitoring apparatus was attached to the participants’ non-dominant arm at their workplace to measure ambulatory blood pressure during the working day. At 16:30 the participants were transported to the Metabolic Unit Research Facility of the North-West University, where they completed a general health questionnaire. At dinnertime they received a standardised meal and had their last beverages at 20:30. They were encouraged to go to bed at around 22:00. After the last blood pressure measurement at 06:00 the next morning, the ambulatory blood pressure monitoring apparatus was removed and subsequent measurements commenced.

### Anthropometric and physical activity measurements

Participants’ height (Invicta Stadiometer, IP 1465, Invicta, London, UK), weight (Precision Health Scale, A & D Company, Tokyo, Japan) and waist circumference (Holtain unstretchable flexible 7 mm wide metal tape) were measured, while wearing minimal clothing, and body mass index (BMI) was calculated, using standard procedures [13]. The mean of triplicate anthropometric measurements was determined. The validated Actical® (Mini Mitter, Bend OR, Montréal, Québec), an omnidirectional accelerometer monitor was worn around the waist and total energy expenditure (TEE) for 24 h taking the basal metabolic rate into account was calculated [14–17]. The basal metabolic rate was estimated from the age, gender, height, and weight of the participants.

### Cardiovascular measurements

The ambulatory blood pressure monitoring device (Meditech Cardiotens CE120®, Budapest, Hungary) measured blood pressure at 30 min intervals during the day (07:00 – 22:00) and one hour intervals during the nighttime (22:00 – 06:00) [18]. Participants were asked to continue with normal daily activities and record any abnormalities such as headache, nausea and stress on their ambulatory diary cards. The successful inflation rate across the 24-h period was 78.8 %. Data obtained was entered into a database using the CardioVisions 1.19 Personal Edition (Meditech, Budapest, Hungary). Hypertensive status was classified from the mean ambulatory blood pressure measurements over a 24 h period as SBP and/or diastolic blood pressure (DBP) ≥130/80 according to the 2013 Guidelines of the European Society of Hypertension/European Society of Cardiology [19]. The carotid dorsalis pedis PWV was measured, on the left side of the participant in the supine position, with the Complior SP Acquisition system (Artech-Medical, Pantin, France). The validated Finometer device (FMS, Finapres Medical Systems, Amsterdam, Netherlands) [20, 21] was used to measure mean arterial pressure and TPR was computed with the model flow method from the pressure waveform [22].

### Biochemical analyses

Blood samples were obtained from each participant by a registered nurse from the antebrachial vein branches, after an overnight fasting period. CK activity, C-reactive protein, total cholesterol, high-density lipoprotein cholesterol, triglycerides and γ-GT levels were determined in serum, while glucose levels were determined in sodium fluoride plasma with the Unicel DXC 800, (Beckman and Coulter, Germany) and the Konelab^TM^ 20I Sequential Multiple Analyzer Computer, (Thermo Scientific, Vantaa, Finland). Both the intra- and inter-assay coefficients of variation for all the assays were less than 10 %. Serum cotinine levels were determined with a homogeneous immunoassay (Automated Modular, Roche, Basel, Switzerland). Ethylenediaminetetraacetic acid treated whole blood was used to determine glycated hemoglobin (Integra 400, Roche, Basel, Switzerland).

### Statistical analyses

Statistica version 12 (Statsoft Inc., Tulsa, OK, USA) was used to perform the statistical analyses of this study. In agreement with our aims, groups were stratified according to gender and ethnicity. Data was expressed as arithmetic mean and standard deviation for normal distributed variables. Variables with a non-Gaussian distribution were logarithmically transformed (CK activity, C-reactive protein, TPR and γGT) and the central tendency and spread represented by the geometric mean and the 5^th^ and 95^th^ percentile intervals. Means and proportions were compared between the ethnic groups, using independent T-tests and Chi-square tests, respectively. Analyses of covariance were also performed while adjusting for, age, BMI and TEE, with PWV additionally adjusted for mean arterial pressure [23]. Associations between cardiovascular variables (blood pressure, PWV and TPR), cardiovascular risk factors (age, lipids, glycated hemoglobin, BMI, cotinine, γ-GT and TEE) and CK activity were investigated in black and white men as well as black and white women, using single and partial regression analysis while adjusting for age and BMI, while PWV was additionally adjusted for mean arterial pressure. In multivariable analyses, we investigated independent associations of ambulatory SBP, PP and TPR with CK activity, while adjusting for traditional (age, total cholesterol and glycated hemoglobin) and behavioural (BMI, cotinine, γ-GT and total energy expenditure) cardiovascular disease risk factors in a forward stepwise procedure. Covariates for entry in multiple regression models were selected based on their univariate associations with markers of cardiovascular function (SBP, PP and TPR) or CK activity. Outliers were identified (>2 x sigma) in residual analyses in a case-wise plot of outliers, and multiple regression analyses repeated. Random distributions around the horizontal axis of residual plots indicated that regression models were appropriate. The required sample size for each group to reach a power (1 – β) of 0.80 were determined for multiple regression models with 8 predictors, with a priori power analyses (G*power v3.1.9.2) [24]. The effect sizes (f^2^) were determined from the partial regression *R*^2^ values and the significance level (α) was set at 0.05. In sensitivity analyses we repeated multiple regression analyses while excluding human immunodeficiency virus infected participants (*n* = 19) and participants on anti-hypertensive medication (*n* = 61). We also repeated multiple regression analyses in normotensive and hypertensive sub-groups separately. We further tested whether associations found in women were dependent on hormonal levels, by adding estradiol as a covariate in multiple regression models.

## Results

### Characteristics of the study population

The characteristics of the study population are represented in Table 1. The black men had a more detrimental cardiovascular profile when compared to the white men, as can be seen from their significantly higher SBP, DBP, and PWV (all *p* < 0.001). CK activity did not differ between black and white men, whereas lifestyle risk factors such as γGT levels were higher, while physical activity was lower in the black men (both *p* < 0.001).

**Table 1:** Characteristics of the study population

	Black men	White men	Black women	White women
*n*	100	101	97	107
Age, years	43.3 (SD 8.11)	45.1 (SD 11.0)	45.7 (SD 7.91)	45.0 (SD 10.8)
Anthropometric measurements				
Height, m	1.70 (SD 0.06)	1.81 (SD 0.07)*	1.59 (SD 0.06)	1.67 (SD 0.06)*
Weight, kg	80.0 (SD 17.8)	95.4 (SD 17.6)*	82.6 (SD 19.0)	72.4 (SD 16.9)*
BMI, kg/m^2^	27.5 (SD 5.76)	29.0 (SD 5.20)*	32.8 (SD 7.23)	26.0 (SD 5.60)*
WC, cm	93.5 (SD 15.5)	102 (SD 14.4)*	93.6 (SD 15.6)	84.7 (SD 13.0)*
Cardiovascular measurements				
SBP, mmHg	137 (SD 16.0)	128 (SD 10.4)*	129 (SD 15.2)	120 (SD 12.4)*
DBP, mmHg	88 (SD 10.8)	80 (SD 7.44)*	79 (SD 8.66)	74 (SD 7.70)*
PP, mmHg	50 (SD 8.31)	48 (SD 6.98)	50 (SD 9.76)	46 (SD 7.63)*
HR, beats/min	79.2 (SD 11.4)	72.0 (SD 11.1)*	80.3 (SD 10.1)	75.0 (SD 8.80)*
PWV, ms	9.20 (SD 2.35)	8.59 (SD 1.35)*	8.21 (SD 1.38)	7.51 (SD 1.30)*
TPR, mmHg/ml/s	1.03 (0.63; 1.78)	0.98 (0.65; 1.62)	0.90 (0.56; 1.51)	0.98 (0.60; 1.66)
Biochemical analyses				
CK activity, U/l	127 (42.7; 427)	115 (46.8; 245)	75.9 (26.9; 195)	62.8 (30.2; 123)*
HbA1c, %	6.24 (SD 1.23)	5.66 (SD 0.48)*	5.91 (SD 1.14)	5.37 (SD 0.29)*
TC, mmol/l	4.74 (SD 1.17)	5.58 (SD 1.20)*	4.44 (SD 1.20)	5.50 (SD 1.35)*
HDL-C, mmol/l	1.05 (SD 0.37)	1.00 (SD 0.27)	1.20 (SD 0.31)	1.40 (SD 0.42)*
TG, mmol/l	1.82 (SD 1.59)	1.51 (SD 0.87)	1.04 (SD 0.64)	0.90 (SD 0.48)
CRP, mg/l	2.73 (0.28; 15.1)	1.80 (1.00; 7.94)*	7.14 (0.78; 35.5)	2.23 (0.99; 14.5)*
Lifestyle and medication				
Cotinine, ng/ml	35 (SD 65.2)	30.9 (SD 96.7)	18.9 (SD 55.7)	15.2 (SD 53.2)
γ-GT, U/l	62.7 (23.4; 331)	27.3 (11.0; 89.1)*	34.9 (16.6; 100)	13.8 (6.17; 38.9)*
TEE, kcal/day	2702 (SD 793)	3674 (SD 2059)*	2652 (SD 797)	2567 (SD 612)
HT, n	79	70	56	31*
HIV infected, n	13	0*	6	0*
Statins, n	1	6	1	3
HT medication, n	20	9*	23	9*

Data expressed as arrhythmic mean (standard deviation), geometric mean (5^th^ and 95^th^ percentiles) or *n*

*SD* standard deviation, *BMI* body mass index, *WC* waist circumference, *SBP* systolic blood pressure, *DBP* diastolic blood pressure, *PP* pulse pressure, *HR* heart rate, *PWV* pulse wave velocity, *TPR* total peripheral resistance, *CK* creatine kinase, *HbA1c* glycated hemoglobin, *TC* total cholesterol, *HDL-C* high density lipoprotein cholesterol, *CRP* C-reactive protein, *γ-GT* γ-glutamyl transferase, *TEE* total energy expenditure, *HT* hypertensive, *HIV* human immunodeficiency virus

*p < 0.05 between black and white men and between black and white women

When comparing black and white women, the black women displayed a worse cardiovascular profile (higher SBP, DBP, PP and PWV, *p* ≤ 0.008). Additionally, the black women also displayed higher mean BMI, waist circumference, CK activity and γ-GT levels (*p* ≤ 0.009).

### Analysis of covariance

The unfavourable cardiovascular profile in the black men and women remained after adjusting for confounding factors (age, BMI and TEE) (Additional file 1: Table S1). There was still no difference in CK activity between black and white men (*p* = 0.21), whereas in black women CK activity (*p* = 0.009) was still significantly higher when compared to the white women.

### Single and partial regression analyses

In single regression analyses (Table 2) significant positive associations between SBP and CK activity (*r* = 0.29; *p* = 0,002) and between PP and CK activity (*r* = 0.29; *p* = 0.002) were indicated only in the white women. After adjusting for age and BMI these associations remained, whereas a significant positive relationship between TPR and CK activity (*r* = 0.24; *p* = 0.02) emerged in the white men. None of these associations were evident in either the black men or women, with the exception of a borderline significant (*r* = 0.20; *p* = 0.056) relationship between TPR and CK activity in the black men, in partial regression analyses.

**Table 2:** Single and partial regression analyses of CK activity with ambulatory blood pressure, pulse wave velocity and total peripheral resistance

	CK activity, U/l							
	Men (*n* = 201)				Women (*n* = 204)			
	Black men (*n* = 100)		White men (*n* = 101)		Black women (*n* = 97)		White women (*n* = 107)	
	Single	Partial	Single	Partial	Single	Partial	Single	Partial
SBP, mmHg	*r* = 0.08	*r* = 0.02	*r* = 0.15	*r* = −0.04	*r* = −0.06	*r* = −0.04	***r*** **= 0.29**	***r*** **= 0.23**
	*p* = 0.43	*p* = 0.84	*p* = 0.14	*p* = 0.73	*p* = 0.59	*p* = 0.65	***p*** **= 0.002**	***p*** **= 0.02**
DBP, mmHg	*r* = 0.03	*r* = −0.03	*r* = 0.12	*r* = −0.01	*r* = 0.03	*r* = 0.04	*r* = 0.18	*r* = 0.10
	*p* = 0.75	*p* = 0.80	*p* = 0.25	*p* = 0.93	*p* = 0.75	*p* = 0.68	*p* = 0.07	*p* = 0.33
PP, mmHg	*r* = 0.11	*r* = 0.07	*r* = 0.10	*r* = −0.04	*r* = −0.11	*r* = −0.11	***r*** **= 0.29**	***r*** **= 0.23**
	*p* = 0.27	*p* = 0.47	*p* = 0.32	*p* = 0.70	*p* = 0.26	*p* = 0.27	***p*** **= 0.002**	***p*** **= 0.02**
PWV, ms	*r* = −0.10	*r* = −0.15	*r* = −0.11	*r* = −0.07	*r* = −0.12	*r* = −0.14	*r* = 0.11	*r* = −0.05
	*p* = 0.32	*p* = 0.17	*p* = 0.26	*p* = 0.51	*p* = 0.25	*p* = 0.19	*p* = 0.27	*p* = 0.61
TPR, mmHg/ml/s	*r* = 0.09	*r* = 0.20	*r* = 0.08	***r*** **= 0.24**	*r* = 0.04	*r* = 0.06	*r* = 0.14	*r* = 0.18
	*p* = 0.38	*p* = 0.056	*p* = 0.42	***p*** **= 0.02**	*p* = 0.70	*p* = 0.56	*p* = 0.15	*p* = 0.08

Relationships adjusted for age and body mass index. Pulse wave velocity was additionally adjusted for mean arterial pressure

*SBP* systolic blood pressure, *DBP* diastolic blood pressure, *PP* pulse pressure, *PWV* pulse wave velocity, *TPR* total peripheral resistance

Significant associations indicated with bold text

### Multivariable analyses

In forward stepwise multiple regression analyses the positive associations of ambulatory SBP and CK activity (adjusted *R*^2^ = 0.46; β = 0.17; *p* = 0.03) and of PP and CK activity (adjusted *R*^2^ = 0.31; β = 0.21; *p* = 0.01) in the white women (Table 3) as well as the positive association of TPR and CK activity (adjusted *R*^2^ = 0.32; β = 0.25; *p* = 0.009) (Table 3) in the white men, remained significant. In a priori power analyses we determined the required sample size for each group to reach a power (1 – β) of 0.80 (black men: *n* = 47; white men: *n* = 33; black women: *n* = 82; white women: *n* = 107).

**Table 3:** Forward stepwise multiple regression analyses

	Model 1: Total peripheral resistance, mmHg/ml/s			
	Black men (*n* = 95)		White men (*n* = 99)	
Adjusted *R* ^2^	0.19		0.32	
	β (95 % CI)	*P*-value	β (95 % CI)	*P*-value
CK activity, log U/l	0.13 (−0.06; 0.34)	0.18	0.25 (0.07; 0.44)	0.009
Age, years	0.19 (−0.004; 0.39)	0.06	0.19 (0.02; 0.36)	0.03
BMI, kg/m^2^	−0.45 (−0.65; −0.23)	<0.001	−0.45 (−0.63; −0.27)	<0.001
γ-GT, log U/l	−0.10 (−0.30; 0.09)	0.31	−0.18 (−0.36; −0.002)	0.049
TC, mmol/l	−0.13 (−0.33; 0.06)	0.18	0.21 (0.03; 0.38)	0.02
TEE, kcal	-	-	−0.13 (−0.30; 0.04)	0.15
	Model 2: Systolic blood pressure, mmHg			
	Black women (*n* = 93)		White women (*n* = 104)	
Adjusted *R* ^2^	0.20		0.46	
	β (95 % CI)	*P*-value	β (95 % CI)	*P*-value
CK activity, log U/l	-	-	0.17 (0.022; 0.31)	0.03
Age, years	0.26 (0.06; 0.45)	0.01	0.22 (0.06; 0.38)	0.03
BMI, kg/m^2^	-	-	0.29 (0.04; 0.54)	0.02
Cotinine	−0.16 (−0.35; 0.03)	0.11	0.13 (−0.02; 0.27)	0.10
HbA1c	0.25 (0.05; 0.45)	0.02	0.22 (0.06; 0.37)	0.008
TEE	0.26 (0.06; 0.45)	0.01	0.26 (0.01; 0.50)	0.04
γ-GT, log U/l	−0.12 (−0.31; 0.07)	0.24	-	-
	Model 3: Pulse pressure, mmHg			
	Black women (*n* = 94)		White women (*n* = 105)	
Adjusted *R* ^2^	0.26		0.31	
	β (95 % CI)	*P*-value	β (95 % CI)	*P*-value
CK activity, log U/l	-	-	0.21 (0.05; 0.38)	0.01
Age, years	0.26 (0.07; 0.44)	0.008	0.21 (0.04; 0.37)	0.02
BMI, kg/m^2^	0.19 (−0.05; 0.43)	0.13	0.46 (0.30; 0.62)	<0.001
Cotinine	-	-	0.14 (−0.02; 0.31)	0.10
HbA1c	0.24 (0.06; 0.43)	0.01	-	-
TEE	0.15 (−0.09; 0.38)	0.24	-	-

Variables included in the models were CK activity, age, BMI, cotinine, γ-GT, HbA1c, total cholesterol and TEE

-, variable did not enter the model, *CK* creatine kinase, *BMI* body mass index, *γ-GT* γ-glutamyl transferase, *TC* total cholesterol, *TEE* total energy expenditure, *HbA1c* glycated hemoglobin

### Sensitivity analyses

After excluding black (*n* = 23) and white women (*n* = 9) as well as black (*n* = 20) and white men (*n* = 9) on anti-hypertensive medication (including statins) the positive associations of ambulatory SBP and CK activity (*R*^2^ = 0.48; β = 0.15; *p* = 0.047) and PP and CK activity (*R*^2^ = 0.28; β = 0.20; *p* = 0.03) in the white women as well as the positive association of TPR and CK activity (*R*^2^ = 0.18; β = 0.24; *p* = 0.02) in the white men remained significant. In normotensive and hypertensive sub-groups the association of TPR and CK activity was only evident in normotensive white men (*R*^2^ = 0.29; β = 0.45; *p* = 0.01), while all the other associations lost significance. Excluding of human immunodeficiency virus infected men (*n* = 13) also did not change the positive association between TPR and CK activity in the white men (*R*^2^ = 0.18; β = 0.22; *p* = 0.03), whereas this association tended to be significant in the black men (*R*^2^ = 0.10; β = 0.21; *p* = 0.058). Applying the same exclusion criteria in women (human immunodeficiency virus infected: *n* = 6) also did not change the positive associations between SBP and CK activity (*R*^2^ = 0.45; β = 0.18; *p* = 0.02) or between PP and CK activity (*R*^2^ = 0.27; β = 0.20; *p* = 0.02) in the white women. The addition of estradiol as covariate in multiple regression analyses in women did not change the results.

## Discussion

In our first main finding we indicated that CK activity is significantly higher in black women when compared to white women, whereas no difference was seen when comparing black and white men. The higher CK activity in black women, confirms previous results indicating higher serum [9, 25] and tissue [4] CK activities in black populations when compared to white populations. However we expected the same result when comparing black and white men, and this result therefore contradicts previous findings [26]. We furthermore also expected the difference in CK activity between black and white women to be greater when considering previous results indicated much higher CK activities in black subjects when compared to white subjects [9, 25].

Various factors may have contributed to the higher CK activity in the black women, including age [27, 28], BMI [29, 30] and different levels of exercise [31]. The black and white women of our study were of similar age, with no difference in their physical activity levels, while their mean BMI values were significantly higher. Nevertheless, even when all of these factors were taken into account (adjusting for age, BMI and TEE), CK activity was still higher in the black women. These factors also had no effect on the CK activity of black and white men.

Our second main finding was the absence of a link between CK activity and cardiovascular measures such as SBP, PP and TPR, in the black men and women despite the higher CK activity in the black women and the worse cardiovascular profile of both black groups. This finding is also contradictory to previous results which indicated blood pressure to increase with increasing CK activity in a multi-ethnic group [6]. We therefore expected to find this link especially in the black men and women. However, as mentioned previously the CK activity in these black participants were not as high as previously reported [9, 25] and other factors may therefore be more important contributors to the worse cardiovascular profile in the black population of our study. Additionally, it has been proposed that the blood pressure-CK activity link is not causal but may be the result of different physiological and metabolic properties between different types of skeletal muscle fibers [32]. Previous results indicated that black participants had less type I [26] and more type II muscle fibers [33]. This phenotype has been linked with obesity [33] and insulin resistance [34] and is further supported by the recent findings of a longitudinal study in which BMI attenuated the predictive value of CK activity in the development of hypertension [30].

Previous findings in the black participants of the SABPA study identified glucose levels [35, 36], the albumin-to-creatinine ratio [37], calcium levels [38], D-dimer levels [39], reactive oxygen species [40] and L-carnitine levels [41] to be positively associated with blood pressure, whereas negative associations of blood pressure with factors such as insulin like growth factor-1 [42] and 8-oxo-7,8-dihydro-2’-deoxyguanosine [43] were also indicated. In a recent longitudinal study, the Prospective Urban and Rural Epidemiology (PURE) study (South-African leg), it was indicated that behavioural risk factors such as γ-GT and abdominal obesity are the main factors contributing to the increase in hypertension incidence [2], with γ-GT levels also predicting cardiovascular and all-cause mortality [10]. Furthermore, psychological distress also predicted the development of hypertension over 5 years [11]. It therefore seems as if the link between higher CK activity and the worse cardiovascular profile in this black population is overshadowed by other contributing factors.

In our final main finding we indicated that TPR are related to CK activity in white men, whereas associations of ambulatory SBP and PP with CK activity were demonstrated in white women. As already mentioned previous studies also indicated a positive relationship between SBP and CK activity in a mixed ethnicity population [6] as well as a normal Norwegian white population [7]. Almost half (48.6 %) of the white participants in our study were hypertensive, which in part may also explain the observed associations of SBP, PP and TPR with CK activity in the white groups, since higher blood pressure may have caused cardiovascular muscle damage and thereby increased the serum CK activity. However, since hypertensive patients exhibits normal iso-enzyme spectra [44], this explanation is considered to be unlikely [6]. Furthermore, results from our study indicated that the association between TPR and CK activity was only evident in normotensive white men in subgroup analyses. The link between blood pressure and CK activity may therefore be explained via two mechanisms [6]. The first mechanism involves enhanced smooth muscle cell contractility as a result of higher availability of cellular energy [5, 6], whereas the second mechanism involves attenuated nitric oxide synthesis as a result of decreased availability of the substrate L-arginine, which is also a substrate for the synthesis of creatine [45]. This mechanism seems very plausible since we previously indicated decreased nitric oxide synthesis capacity in these white men and women [46]. Additionally, it has been suggested that even a minor increase in the contractility of smooth muscle could have a vast effect on the resistance of blood flow [6], which may in turn explain the TPR-CK activity link observed in the white men of this study.

This study has to be interpreted within the context of its limitations and strengths. This was a cross-sectional study and we can therefore not infer causality. We did not assess iso-enzymes in this study, which may be important to clarify the mechanisms involved. Although our population size is rather small when compared to other studies, power calculations indicated the population size to be sufficient. Our study participants were not asked to rest for three days before blood collection for CK activity analysis but we adjusted for physical activity measured during the 24 h prior to blood collection.

## Conclusions

In conclusion, we have indicated higher CK activity in black women when compared to white women, with no difference when comparing the men. We were unable to establish a link between blood pressure or TPR and CK activity in black men or women, suggesting that other factors such as increased behavioural risk factors related to rapid urbanization may be more important contributing risk factors to the high prevalence of cardiovascular disease in this population. Whereas, the established link between cardiovascular function and CK activity in the white population may be the result of enhanced smooth muscle cell contractility and/or attenuated nitric oxide synthesis capacity.

## Abbreviations

BMI, body mass index; CK, creatine kinase, DBP, diastolic blood pressure, PP, pulse pressure, PURE, Prospective Urban and Rural Epidemiology, PWV, pulse wave velocity, SABPA, Sympathetic activity and Ambulatory Blood Pressure in Africans, SBP, systolic blood pressure, TEE, total energy expenditure, TPR, total peripheral resistance, γ-GT, γ-glutamyl transferase

## Additional file

Additional file 1: Table S1. Analyses of covariance. (DOC 39 kb)

## References

[1] Van Rooyen J, Kruger H, Huisman H, Wissing M, Margetts B, Venter C, Vorster H (2000). An epidemiological study of hypertension and its determinants in a population in transition: the THUSA study. J Hum Hypertens.

[2] Schutte AE, Schutte R, Huisman HW, Van Rooyen JM, Fourie CM, Malan NT, Malan L, Mels CM, Smith W, Moss SJ (2012). Are behavioural risk factors to be blamed for the conversion from optimal blood pressure to hypertensive status in Black South Africans? A 5-year prospective study. Int J Epidemiol.

[3] Sliwa K, Wilkinson D, Hansen C, Ntyintyane L, Tibazarwa K, Becker A, Stewart S (2008). Spectrum of heart disease and risk factors in a black urban population in South Africa (the Heart of Soweto Study): a cohort study. The Lancet.

[4] Brewster LM, Coronel CM, Sluiter W, Clark JF, Van Montfrans GA (2012). Ethnic differences in tissue creatine kinase activity: an observational study. PLoS One.

[5] Brewster LM, Clark JF, van Montfrans GA (2000). Is greater tissue activity of creatine kinase the genetic factor increasing hypertension risk in black people of sub‐Saharan African descent?. J Hypertens.

[6] Brewster LM, Mairuhu G, Bindraban NR, Koopmans RP, Clark JF, van Montfrans GA (2006). Creatine kinase activity is associated with blood pressure. Circulation.

[7] Johnsen SH, Lilleng H, Wilsgaard T, Bekkelund SI (2011). Creatine kinase activity and blood pressure in a normal population: the Tromsø study. J Hypertens.

[8] Watanabe M, Okamura T, Kokubo Y, Higashiyama A, Okayama A (2009). Elevated serum creatine kinase predicts first-ever myocardial infarction: a 12-year population-based cohort study in Japan, the Suita study. Int J Epidemiol.

[9] Wong E, Cobb C, Umehara M, Wolff G, Haywood L, Greenberg T, Shaw S (1983). Heterogeneity of serum creatine kinase activity among racial and gender groups of the population. Am J Clin Pathol.

[10] Zatu MC, Van Rooyen JM, Kruger A, Schutte AE. Alcohol intake, hypertension development and mortality in black South Africans. Eur J Prev Cardiol. 2016;23(3):308–15. 10.1177/2047487314563447 25500903

[11] Schutte AE, Ware LJ, Huisman HW, Fourie CM, Greeff M, Khumalo T, Wissing MP. Psychological Distress and the Development of Hypertension Over 5 Years in Black South Africans. J Clin Hypertens. 2014;2:126–33. 10.1111/jch.12455 PMC8031906 25496159

[12] Malan L, Hamer M, Frasure-Smith N, Steyn HS, Malan NT. COHORT PROFILE: Sympathetic activity and Ambulatory Blood Pressure in Africans (SABPA) Prospective Cohort Study. Int J Epidemiol. 2015;44(6):1814-22. 10.1093/ije/dyu199 PMC4689997 25344943

[13] Marfell-Jones MJ, Stewart A, de Ridder J (2012). International standards for anthropometric assessment.

[14] Heil DP (2006). Predicting activity energy expenditure using the Actical® activity monitor. Res Q Exerc Sport.

[15] Dannecker KL, Sazonova NA, Melanson EL, Sazonov ES, Browning RC (2013). A comparison of energy expenditure estimation of several physical activity monitors. Med Sci Sports Exerc.

[16] Dugas L, Carstens M, Ebersole K, Schoeller D, Durazo-Arvizu R, Lambert E, Luke A (2009). Energy expenditure in young adult urban informal settlement dwellers in South Africa. Eur J Clin Nutr.

[17] Dugas LR, Bovet P, Forrester TE, Lambert EV, Plange-Rhule J, Durazo-Arvizu RA, Shoham D, Kroff J, Cao G, Cooper RS (2014). Comparisons of intensity-duration patterns of physical activity in the US, Jamaica and 3 African countries. BMC Public Health.

[18] Kohara K, Nishida W, Maguchi M, Hiwada K (1995). Autonomic nervous function in non-dipper essential hypertensive subjects evaluation by power spectral analysis of heart rate variability. Hypertension.

[19] Mancia G, Fagard R, Narkiewicz K, Redán J, Zanchetti A, Böhm M, Christiaens T, Cifkova R, De Backer G, Dominiczak A (2013). 2013 Practice guidelines for the management of arterial hypertension of the European Society of Hypertension (ESH) and the European Society of Cardiology (ESC): ESH/ESC Task Force for the Management of Arterial Hypertension. J Hypertens.

[20] Imholz BP, Wieling W, van Montfrans GA, Wesseling KH (1998). Fifteen years experience with finger arterial pressure monitoring: assessment of the technology. Cardiovasc Res.

[21] Guelen I, Westerhof BE, van der Sar GL, van Montfrans GA, Kiemeneij F, Wesseling KH, Bos WJW (2008). Validation of brachial artery pressure reconstruction from finger arterial pressure. J Hypertens.

[22] Wesseling K, Jansen J, Settels J, Schreuder J (1993). Computation of aortic flow from pressure in humans using a nonlinear, three-element model. J Appl Physiol.

[23] Collaboration RVfAS (2010). Determinants of pulse wave velocity in healthy people and in the presence of cardiovascular risk factors: ‘establishing normal and reference values’. Eur Heart J.

[24] Faul F, Erdfelder E, Lang A-G, Buchner A (2007). G* Power 3: A flexible statistical power analysis program for the social, behavioral, and biomedical sciences. Behav Res Methods.

[25] Black HR, Quallich H, Gareleck CB (1986). Racial differences in serum creatine kinase levels. Am J Med.

[26] Ama P, Simoneau J, Boulay M, Serresse O, Theriault G, Bouchard C (1986). Skeletal muscle characteristics in sedentary black and Caucasian males. J Appl Physiol.

[27] Swaminathan R, Ho C, Donnan S (1988). Body composition and plasma creatine kinase activity. Ann Clin Biochem.

[28] Neal RC, Ferdinand KC, Yčas J, Miller E (2009). Relationship of ethnic origin, gender, and age to blood creatine kinase levels. Am J Med.

[29] Salvadori A, Fanari P, Ruga S, Brunani A, Longhini E. Creatine kinase and creatine kinase-MB isoenzyme during and after exercise testing in normal and obese young people. Chest. 1992;102(6):1687–9. 10.1378/chest.102.6.1687 1446472

[30] Johnsen SH, Lilleng H, Bekkelund SI. Creatine Kinase as Predictor of Blood Pressure and Hypertension. Is It All About Body Mass Index? A Follow‐Up Study of 250 Patients. J Clin Hypertens. 2014;16(11):820–6. 10.1111/jch.12422 PMC8031622 25279588

[31] Nicholson GA, McLeod JG, Morgan G, Meerkin M, Cowan J, Bretag A, Graham D, Hill G, Robertson E, Sheffield L (1985). Variable distributions of serum creatine kinase reference values: relationship to exercise activity. J Neurol Sci.

[32] Pickering TG. Muscular hypertension: is creatine kinase responsible for hypertension in blacks? J Clin Hypertens. 2008;10(1):73–6. 10.1111/j.1524-6175.2007.07846.x PMC8109889 18174774

[33] Tanner CJ, Barakat HA, Dohm GL, Pories WJ, MacDonald KG, Cunningham PR, Swanson MS, Houmard JA. Muscle fiber type is associated with obesity and weight loss. Am J Physiol Endocrinol Metab. 2002;282(6):E1191–6. 10.1152/ajpendo.00416.2001 12006347

[34] Lillioja S, Young AA, Culter CL, Ivy JL, Abbott W, Zawadzki JK, Yki-Järvinen H, Christin L, Secomb TW, Bogardus C. Skeletal muscle capillary density and fiber type are possible determinants of in vivo insulin resistance in man. J Clin Invest. 1987;80(2):415. 10.1172/JCI113088 PMC442253 3301899

[35] Lammertyn L, Schutte AE, Schutte R (2011). Blood glucose and nocturnal blood pressure in African and Caucasian men: The SABPA study. Diabetes Res Clin Pract.

[36] Lammertyn L, Schutte R, Schutte AE, Huisman HW, van Rooyen JM, Malan NT, Fourie CM, Malan L (2011). Associations of cholesterol and glucose with cardiovascular dysfunction in black Africans: the SABPA study. Clin Exp Hypertens.

[37] Schutte R, Schutte AE, Huisman HW, Glyn MC, van Rooyen JM, Malan NT, Fourie CM, Malan L (2011). Arterial stiffness, ambulatory blood pressure and low-grade albuminuria in non-diabetic African and Caucasian men: the SABPA study. Hypertens Res.

[38] Schutte R, Huisman HW, Schutte AE, Malan NT, van Rooyen JM, Fourie CM, Malan L (2010). Serum calcium revisited: associations with 24-h ambulatory blood pressure and cardiovascular reactivity in Africans. Hypertens Res.

[39] Lammertyn L, Schutte AE, Pieters M, Schutte R (2014). D-dimer relates positively with increased blood pressure in black South Africans: The SABPA study. Thromb Res.

[40] Kruger R, Schutte R, Huisman H, Van Rooyen J, Malan N, Fourie C, Louw R, Van der Westhuizen F, Van Deventer C, Malan L (2012). Associations between reactive oxygen species, blood pressure and arterial stiffness in black South Africans: the SABPA study. J Hum Hypertens.

[41] Mels CM, Schutte AE, Erasmus E, Huisman HW, Schutte R, Fourie CM, Kruger R, Van Rooyen JM, Smith W, Malan NT (2013). L-Carnitine and long-chain acylcarnitines are positively correlated with ambulatory blood pressure in humans: the SABPA study. Lipids.

[42] Schutte A, Schutte R, Smith W, Huisman H, Mels C, Malan L, Fourie C, Malan N, Van Rooyen J, Kruger R (2014). Compromised bioavailable IGF-1 of black men relates favourably to ambulatory blood pressure: The SABPA study. Atherosclerosis.

[43] Mels CM, Schutte AE, Schutte R, Pretorius PJ, Smith W, Huisman HW, van der Westhuizen FH, Fourie CM, van Rooyen JM, Kruger R (2014). 8-Oxo-7, 8-dihydro-2'-deoxyguanosine, reactive oxygen species and ambulatory blood pressure in African and Caucasian men: The SABPA study. Free Radic Res.

[44] Brewster LM, van Bree S, Reijneveld JC, Notermans NC, Verschuren W, Clark JF, van Montfrans GA, de Visser M (2008). Hypertension risk in idiopathic hyperCKemia. J Neurol.

[45] Wu G, Morris J. Arginine metabolism: nitric oxide and beyond. Biochem J. 998;336:1–17. 10.1042/bj3360001 PMC1219836 9806879

[46] Mels CM, Loots I, Schwedhelm E, Atzler D, Böger RH, Schutte AE. Nitric oxide synthesis capacity, ambulatory blood pressure and end organ damage in a black and white population: the SABPA study. Amino Acids. 2016;48:801–10. 10.1007/s00726-015-2128-5 26573539

