# Supplementary material for: Cardiovascular function is not associated with creatine kinase activity in a black African population: The SABPA study
Source: BMC Cardiovasc Disord. 2016 Jun 10;16:134. doi: 10.1186/s12872-016-0315-2 (PMC4902899; doi:10.1186/s12872-016-0315-2)
Supplement: Additional file 1: Table S1. — Analyses of covariance. (DOC 39 kb) [file 12872_2016_315_MOESM1_ESM.doc]

**Table S1** Analyses of covariance

|  | ***Black men*** | ***White men*** | ***Black women*** | ***White women*** |
| --- | --- | --- | --- | --- |
| n | 100 | 101 | 97 | 107 |
| SBP, mmHg | 138 (136; 141) | 127 (124; 129)* | 127 (124; 129) | 122 (119; 124)* |
| DBP, mmHg | 89 (87; 90) | 79( 77; 81)* | 79 (77; 80) | 74 (73; 76)* |
| PP, mmHg | 50 (48; 51) | 48 (47; 50) | 49 (47; 50) | 48 (46; 49)* |
| HR, beats/min | 79.8 (77.5; 82.0) | 71.4 (69.2; 73.6)* | 79.5 (77.4; 81.6) | 75.8 (73.8; 77.8)* |
| PWV^†^, ms | 9.12 (8.74; 9.51) | 8.71 (8.33; 9.08)* | 8.06 (7.80; 8.31) | 7.66 (7.42; 7.90)* |
| TPR, mmHg/ml/s | 1.01 (0.95; 1.07) | 1.00 (0.99; 1.07) | 0.95 (0.89; 1.00) | 0.93 (0.87; 0.98) |
| CK activity, U/l | 132 (117; 148) | 109 (100; 123) | 75.9 (67.6; 85.1) | 63.1 (56.2; 70.8)* |
| γ-GT, U/l | 63.1 (55.0; 72.4) | 26.9 (23.4; 30.9)* | 33.1 (28.8; 38.0) | 14.5 (12.6; 16.6)* |

Data expressed as adjusted means with 5^th^ and 95^th^ confidence intervals

*p<0.05 between black and white men and between black and white women

^†^Additionally adjusted for MAP

MAP, mean arterial pressure; SBP, systolic blood pressure; DBP, diastolic blood pressure; PP, pulse pressure; HR, heart rate; PWV, pulse wave velocity; TPR, total peripheral resistance; CK, creatine kinase; γ-GT, γ-glutamyl transferase.
